# Flexible and compact hybrid metasurfaces for enhanced ultra high field *in vivo* magnetic resonance imaging

**DOI:** 10.1038/s41598-017-01932-9

**Published:** 2017-05-10

**Authors:** Rita Schmidt, Alexey Slobozhanyuk, Pavel Belov, Andrew Webb

**Affiliations:** 1 Department of Radiology, Leiden University Medical Center, Leiden, Netherlands; 2 Department of Nanophotonics and Metamaterials, ITMO University, St. Petersburg, Russia; 3 Nonlinear Physics Center, Australian National University, Canberra, ACT 2601 Australia

## Abstract

Developments in metamaterials and related structures such as metasurfaces have opened up new possibilities in designing materials and devices with unique properties. Here we report a new hybrid metasurface structure, comprising a two-dimensional metamaterial surface and a very high permittivity dielectric substrate, that has been designed to enhance the local performance of an ultra-high field MRI scanner. This new flexible and compact resonant structure is the first metasurface which can be integrated with multi-element close-fitting receive coil arrays that are used for all clinical MRI scans. We demonstrate the utility of the metasurface acquiring *in-vivo* human brain images and proton MR spectra with enhanced local sensitivity on a commercial 7 Tesla system.

## Introduction

Metamaterials offer a unique platform for controlling the propagation of acoustic and electromagnetic waves^1–4^. Developments in metamaterials^5, 6^ and related structures such as metasurfaces^7–10^ have opened up new possibilities in designing materials and devices with unique properties^11, 12^. Most applications of metamaterials have been demonstrated in the optical (tens to hundreds of THz) and microwave (tens to hundreds of GHz) frequency ranges using sub-millimeter-sized unit-cells. One of the potential clinical applications of metamaterials is to magnetic resonance imaging (MRI), one of the most common diagnostic techniques used in hospitals worldwide. Human MRI systems operate over a frequency range of approximately 40–400 MHz, i.e. in the radiofrequency (RF) spectrum. Several studies have shown proof-of-principle implementations of metamaterials in MRI using lenses based on split rings^13^, swiss-rolls^14^, discrete wires^15–17^, magnetoinductive waveguides^18^ and travelling-wave excitation^19^. However, since the size of the unit-cells for metamaterials for RF applications lies in the centimetre to tens-of-centimetre range, the vast majority of these previous implementations are based on three-dimensional metamaterial structures that have very large physical dimensions with respect to the available space within an MRI scanner. This is problematic since all clinical MRI scanners use a large array of RF receive coils which are placed as close to the body as possible for maximum sensitivity. The inclusion of large metamaterial structures mean that the coil array must be placed some distance away from the body and the resulting loss in sensitivity can cancel out much of the theoretical increase from the metamaterial. The large size of these structures means that there are currently no practical implementations of metamaterials on commercial MRI scanners.

One solution to overcome these problems is to design, thin and flexible metasurfaces^7–10^ which can be fully integrated into a multi-element receive array. Metasurfaces enable the control of electromagnetic waves in both linear^20^ and nonlinear^21, 22^ regimes and so can be used to shape the RF field distribution in the region-of-interest (ROI). Initial work has been performed by Algarin *et al*. using a thin (11 mm) metamaterial slab based on split-ring resonators^23^. However, this metamaterial introduces significant extra noise due to the large number of lumped elements present and does not have any flexibility in terms of spatially redistributing the near field magnetic and electric field components. A large, non-flexible metasurface resonator has also recently been shown to increase the local sensitivity of 1.5 T MRI^16^ However, the use of such a structure in real practice is limited, due to the large physical size (tens of centimetres in each dimension) of the metasurface components which prevents them from being able to function with a dedicated receive array.

Our new approach is to design a thin, compact and flexible metasurface which can be placed between the patient and a close-fitting receive coil array. The metasurface is formed using thin conductor strips coupled to a flexible high permittivity pad. We demonstrate the application of such a metasurface in human brain MRI and localized MR spectroscopy at 7 Tesla, concentrating on using the metasurface to produce a local increase in the SNR in the occipital cortex. The very thin metasurface fits into a commercial 32-channel receive array and the results reported in this paper represents the first efficient practical metasurface-based device. It should also be noted that other MRI-based approaches for achieving local increase in the SNR are complementary and can be combined with the metasurface approach. These include spatial control of the RF excitation by pulse design methods such as kT-points and spokes^24, 25^, as well as the use of multiple-transmit parallel excitation^26, 27^. RF field shaping can also be achieved using high permittivity materials^28–30^ or active off-resonant structures^31^. However, these two latter approaches lack the possibility of controlling the exact shape of the local field at subwavelength scales, which is possible using metasurfaces.

## Results

### Electromagnetic modelling and design of the metasurface

**Figure 1:**
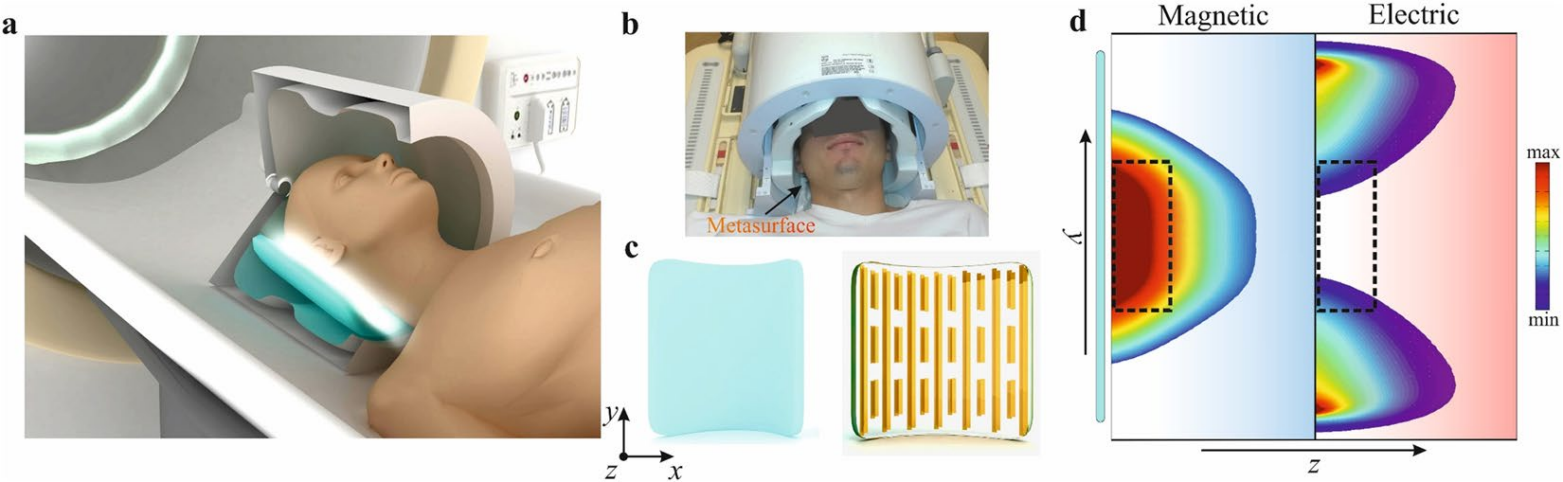
Structural geometry of the metamaterial and simulation of near field magnetic and electric field distributions. (**a**) Schematic of the MRI setup with a cut-out for better visualization of the setup. (**b**) A photograph of the *in-vivo* experiment including the transmit (outer) and multi-element receive coil array (inner). (**c**) Artist’s view of the hybrid metasurface, including high permittivity dielectric substrate (left) combined with its metallic structure (right). (**d**) Numerically calculated magnetic (left) and electric (right) field maps in vacuum near the metasurfaces (shown as a blue rectangle). The region of interest is depicted as a black dashed rectangle.

The new hybrid metasurface is shown schematically in Fig. 1. Metallic strips with a thickness of a few tens of micrometres are attached to both sides of a flexible 8 mm thick pad made from a CaTiO_3_ suspension in water with a relative permittivity of 110. In a typical setup for human brain imaging there is approximately a 1–2 cm distance between the array and the subject’s head. The metasurface is designed to fit between the patient and the close-fitting RF receive coil array (Fig. 1a). The metasurface can be placed next to any region of the brain: in this demonstration we targeted the occipital cortex, which is commonly studied in anatomical, functional and spectroscopic studies of the visual cortex.

The fundamental principle behind the magnetic field enhancement is the spatial redistribution and change in magnitude of electromagnetic near fields by the resonant excitation of the specific eigenmode of the metasurface. This phenomenon can be understood by considering the metamaterial’s geometry (Fig. 1c). The length of the longer strips is designed to be slightly shorter than that required to produce the first half-wavelength resonance at 298 MHz. The length and the spacing between the strips control the frequency of the generated standing waves (or resonant modes) as has been shown in previous studies^15–17^. These resonant modes are produced by the effective negative permittivity generated by the set of long strips in our setup. The specific eigenmode of the metasurface used for MRI has a similar magnetic field distribution to the TE_01_ mode of a square dielectric resonator (see Supporting Information S1 for more details of the eigenmode).

It is interesting to note that an analogous structure has been employed to achieve a negative refractive index^32^ in microwave applications. Figure 1d shows numerically calculated magnetic (left) and electric (right) field maps in the region of interest which is depicted as a dashed rectangle. Placing the metasurface near the patient modifies the field pattern of the RF coil due to the “focusing” of the magnetic field (Fig. 1d) in the region-of-interest (ROI). The “focusing” exploits the passively coupling of the metamaterial to the MR excitation coil. In this way, the metasurface is able to provide higher local efficiency of the RF transmitted field and a higher image SNR due to higher receive sensitivity. It is important to note that, while in the case of negative refraction^32^ the short wire pairs represent an artificial “magnetic atom” which displays magnetic resonance^33^, in the current design we design the non-resonant short strips in order to further modify the near field pattern of the eigenmode to obtain a greater increase in the MRI sensitivity and to perform fine tuning of the metasurface (see Supporting Information S2).

### Phantom simulations and experimental data

**Figure 2:**
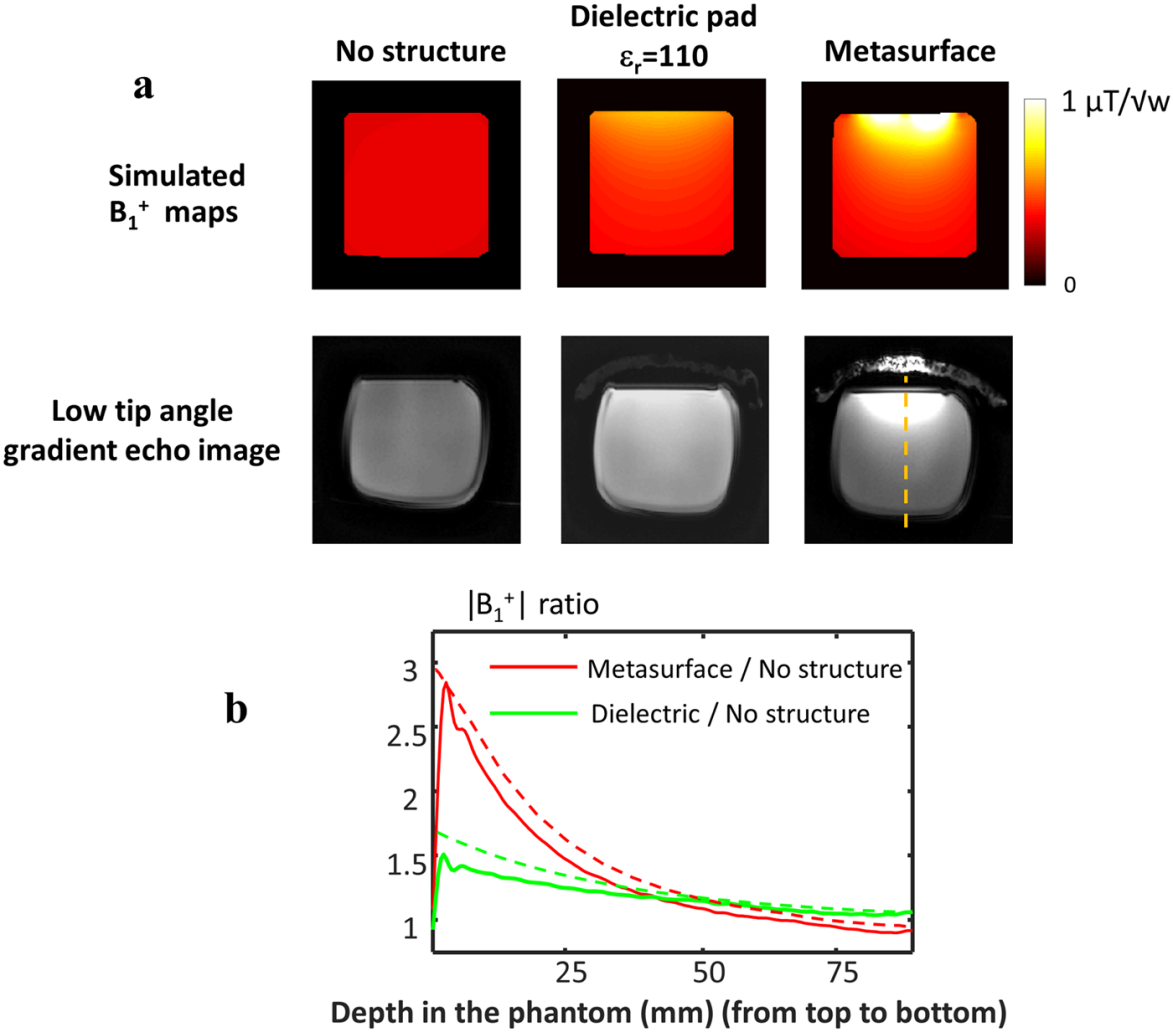
Simulated and measured transmit (B_1_^+^) fields in an oil phantom. (**a**) B_1_^+^ maps and the experimentally acquired low flip angle gradient echo images: (left to right) no artificial structure; with dielectric material only; with the metasurface structure. (**b**) Measured (solid line) and simulated (dashed line) B_1_^+^ profile, along the yellow dashed line. Supporting Information S3 includes an illustration of the experimental setup.

Initial simulations and experimental data were performed using a simple homogenous phantom to determine the correspondence between simulations and experiments. The phantom consisted of a rectangular cuboid filled with mineral oil which has a relative permittivity of 5 and therefore no constructive/destructive wavelength effects occur within the phantom at an operating frequency of 300 MHz. The experiment was performed using a quadrature birdcage coil for both transmit and receive and a low-tip angle excitation gradient echo sequence which produces images in which the SNR is proportional to the product of the RF transmit field (B_1_^+^) and the complex conjugate of the receive sensitivity (B_1_^−*^) divided by the square root of the accepted power (see Methods for more details). Three setups were used – the first with only the phantom, the second with an 8 mm thick CaTiO_3_ dielectric pad placed on top of the phantom and the third with the metasurface structure on top. Figure 2 compares the electromagnetic simulations and the experimentally-acquired images. It can be seen in the simulation that the increase in the magnitude of the B_1_^+^ field is approximately a factor-of-three close to the metasurface. Since the same RF coil is used to receive the signal, the B_1_^−^ also increases by the same factor-of-three by the principle of reciprocity.

### *In vivo* simulations and experimental data

**Figure 3:**
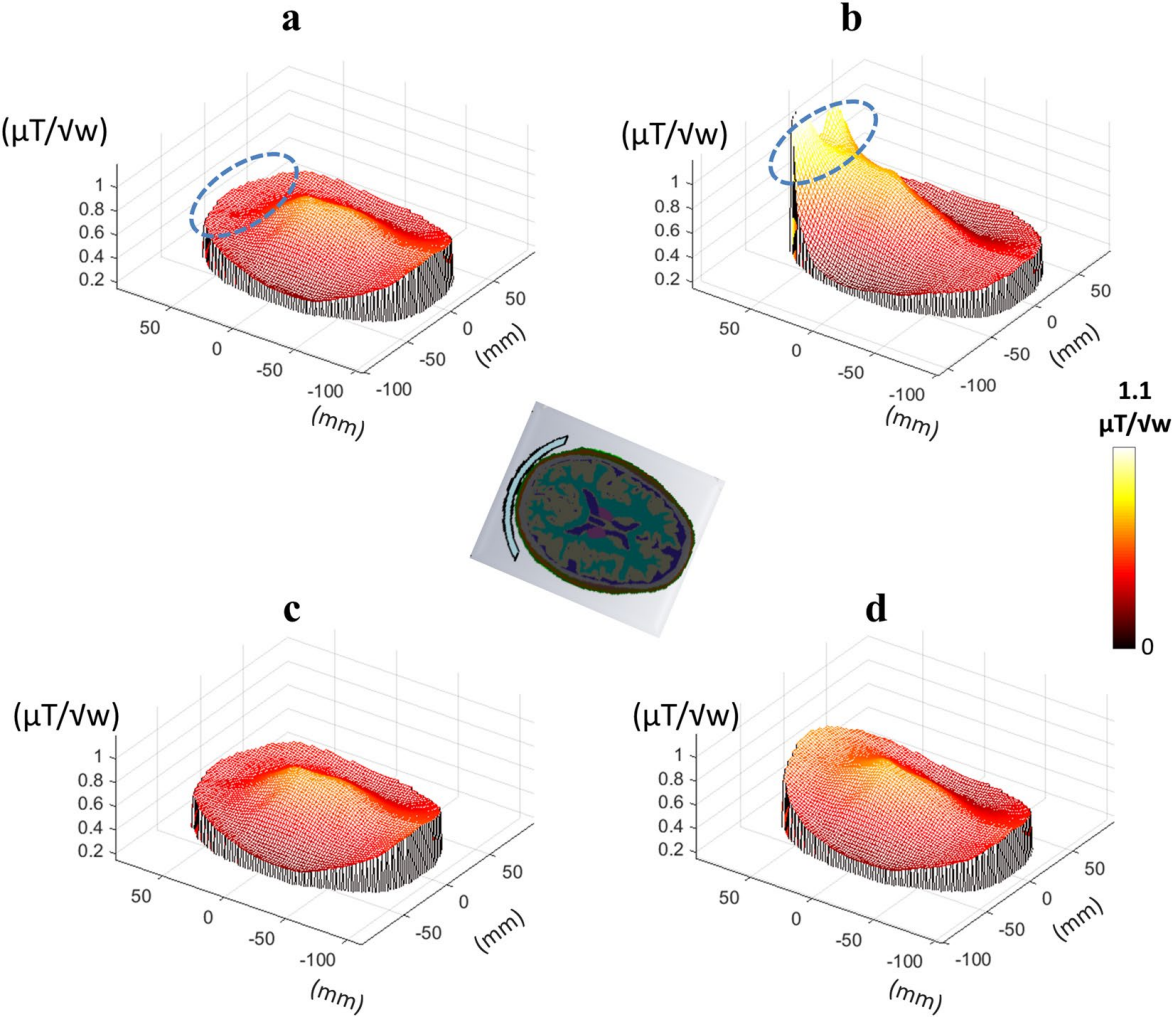
EM simulations of the human head showing mesh plots of the B_1_^+^ distribution along a central axial cross-section of the brain for the following setups: (**a**) no structure, (**b**) with metasurface tuned to 298 MHz, (**c**) with metallic strips only and (**d**) with the dielectric substrate only. The axial cross section of the brain and the metasurface location (in blue) is shown in the centre. The blue dashed ellipse shows the region of interest in the occipital cortex.

Figure 3 compares simulations of the B_1_^+^ field in an electromagnetic model of the human head: (a) under normal scanning conditions, (b) with the metasurface placed close to the occipital cortex, (c) with the copper strips only and (d) with the dielectric material only. The effect of the metasurface is clear, with a maximum enhancement ratio of approximately 2.7 compared to the normal scanning conditions.

*In-vivo* brain scanning was performed with the metasurface structure placed close to the occipital cortex, as shown in Fig. 1a. The same quadrature birdcage coil was used for RF transmission and a close-fitting 32-channel array coil for signal detection. We measured the effect of the metasurface on the isolation between different receiver elements in the 32-channel receive coil array, loaded with a phantom that simulated the human head, via S_12_ measurements of different pairs of elements using a network analyzer. There were no systematic differences, with some channels showing slightly enhanced (~1 dB) coupling and others slightly reduced (~1 dB) coupling. In addition to the magnetic field distribution, it is essential to consider the effect of the metasurface on the power deposition in the patient, which is expressed as the local specific absorption rate (SAR) averaged over 10 grams. Electromagnetic simulations show that the maximum local SAR in the brain was increased from 0.4 W/kg to 0.65 W/kg with the metasurface in place. However, this value lies well below the FDA limit of 3.2 W/kg for *in vivo* imaging.

**Figure 4:**
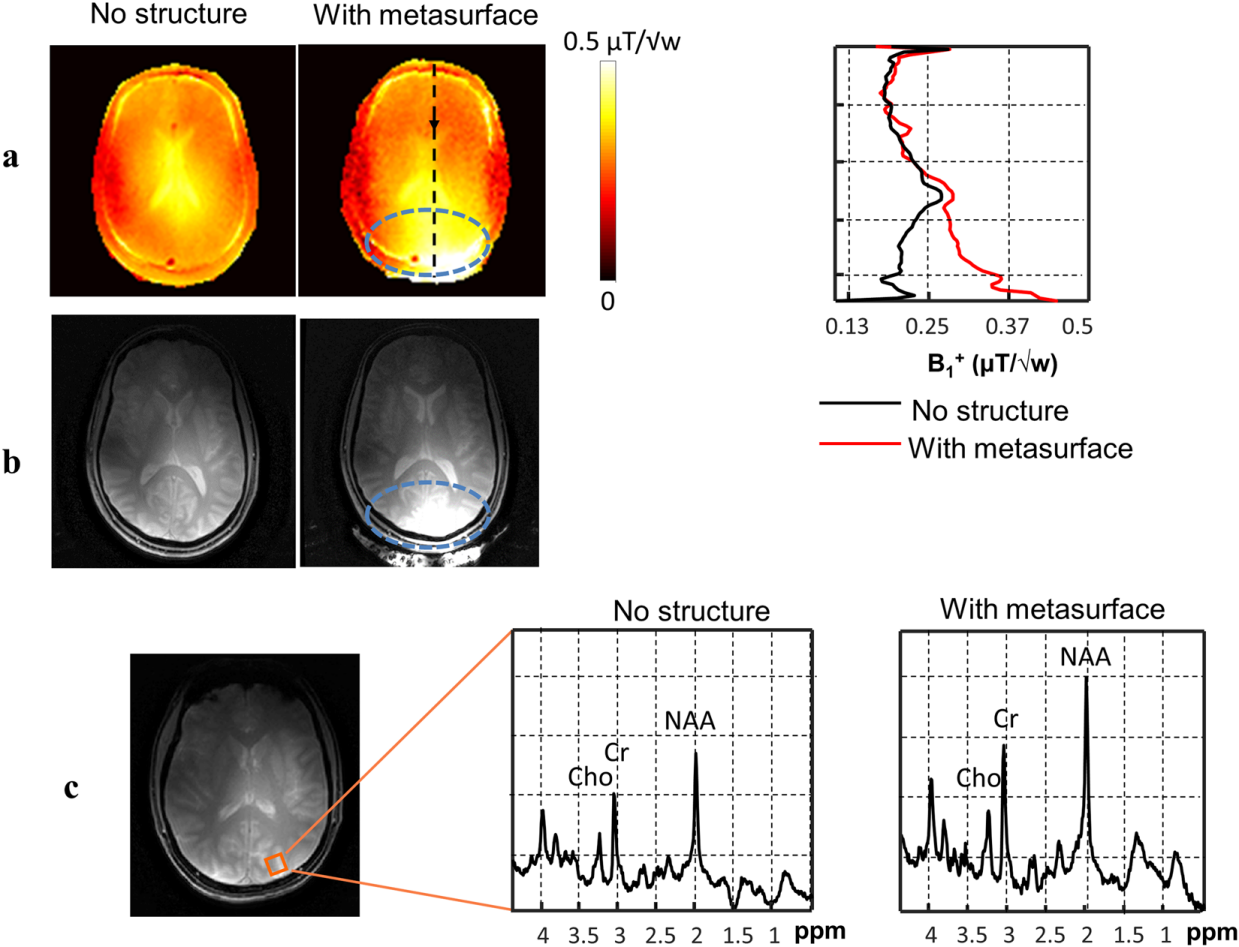
*In-vivo* imaging and spectroscopy results with and without the metasurface. (**a**) Measured RF transmit field (B_1_^+^) maps and B_1_^+^ profile along the dashed black line. (**b**) Low flip angle MR images of the brain. (**c**) Localized ^1^H spectroscopy performed without (left) and with (right) the metasurface (the location of the voxel is shown by the orange overlay in the anatomic image). The intensities of the spectral plots are in arbitrary units (normalized to the root-mean-square noise).

Figure 4 shows experimental maps (in one of the four volunteers studied) of the B_1_^+^ produced using the metasurface close to the occipital cortex: the B_1_^+^ is derived from a low tip angle gradient echo image which is also displayed. Four volunteers were scanned and the average enhancement ratio over the ROI for the RF transmit field was 2.0 ± 0.3. Taking into account the previously-mentioned increase in maximum SAR, the increase in transmit efficiency per square root of maximum SAR, the standard metric used in assessing RF coil efficiency, was a factor of 1.6 ± 0.24. In practical terms the increase in transmit efficiency means that the amplitude or duration of the transmitted RF pulse can be reduced by the equivalent factor.

The average enhancement in the receive field was measured to be 1.9 ± 0.2 (the enhancement in the receive field was normalized by the standard deviation of the noise in the image). The image SNR increases by a factor proportional to *B*_*1*_^*−*^ and in SNR-limited applications this increase can be converted into higher imaging resolution or reduced scanning times. An example of the type of experiment which is very suited to high field, localized ^1^H spectra from a small area in the occipital lobe are shown in Fig. 4c. An increase of 50% in the SNR of the spectra was obtained by using the metasurface, which agrees well with the increase in the simulated B_1_^−^ field integrated over the spectroscopic volume. This increase of 50% corresponds to a decrease in total experimental time of a factor-of-two for a constant SNR, which represents an important improvement for spectroscopic acquisitions which typically take tens of minutes.

## Discussion

This study has demonstrated a new design of a flexible and compact hybrid metasurface structure which can enhance the local RF transmit and receive efficiency in MRI. Since the design is thin and flexible it can be shaped to the anatomy of the patient, which is essential for combined operation with high density receive arrays. The structures enable manipulation of the magnetic field distribution in the region of interest, demonstrating the first applications of metasurfaces-based devices for *in-vivo* imaging and spectroscopy of the brain, concentrating on the occipital cortex as a region of interest. This represents the first practical demonstration of a metasurface integrated with the multi-element receive arrays which are used in all clinical scans. We have shown results at very high field, but this approach can also be used at 3 Tesla and 1.5 Tesla which are currently used for most clinical studies.

Although the flexibility of the substrate has several advantages, bending can potentially shift the frequency of the mode and affect the mutual coupling between the sub-units^34–36^. Therefore, we analyzed the deviation of the resonant mode due to the bending of the structure and compared the H-field and E-field of a flat versus curved metasurface loaded with a phantom with relative permittivity of 45 representing brain tissue at 7 T: the full data are shown in Supporting Information S4. The results show a slight shift in resonance frequency of 5 MHz and an increase of 3% in the peak of the H-field and 7% in the E-field, in a curved versus flat metasurface. There is also the chance of slight differences in the positioning of the metasurface due to the fact that different patients have different head sizes and ellipticity. Supporting Information S5 shows an analysis of such deviations. The results show that local enhancement ratio can deviate in the range of 2–3.

In previous work an increase in the noise due to ohmic losses of a magnetoinductive lens based on split-rings has been reported^23^. However, in our experimental data we did not measure any increase in the noise level (calculated as the standard deviation of the noise in a region outside the phantom). This could be due to several reasons including the fact that no lossy lumped elements are required in this design compared to the split-ring implementation^23, 35^. In addtion, losses and associated noise from a conductive sample are higher at 7 T than at the lower fields where split-rings have been used, since the conductivity of human tissue increases with frequency. We also measured the effect on the SNR of the metallic strips alone by replacing the high permittivity material with a plastic layer with permittivity of 1. These SNR measurements were repeated and no SNR change was observed due to the metallic strips (see Supporting Information S6).

**Figure 5:**
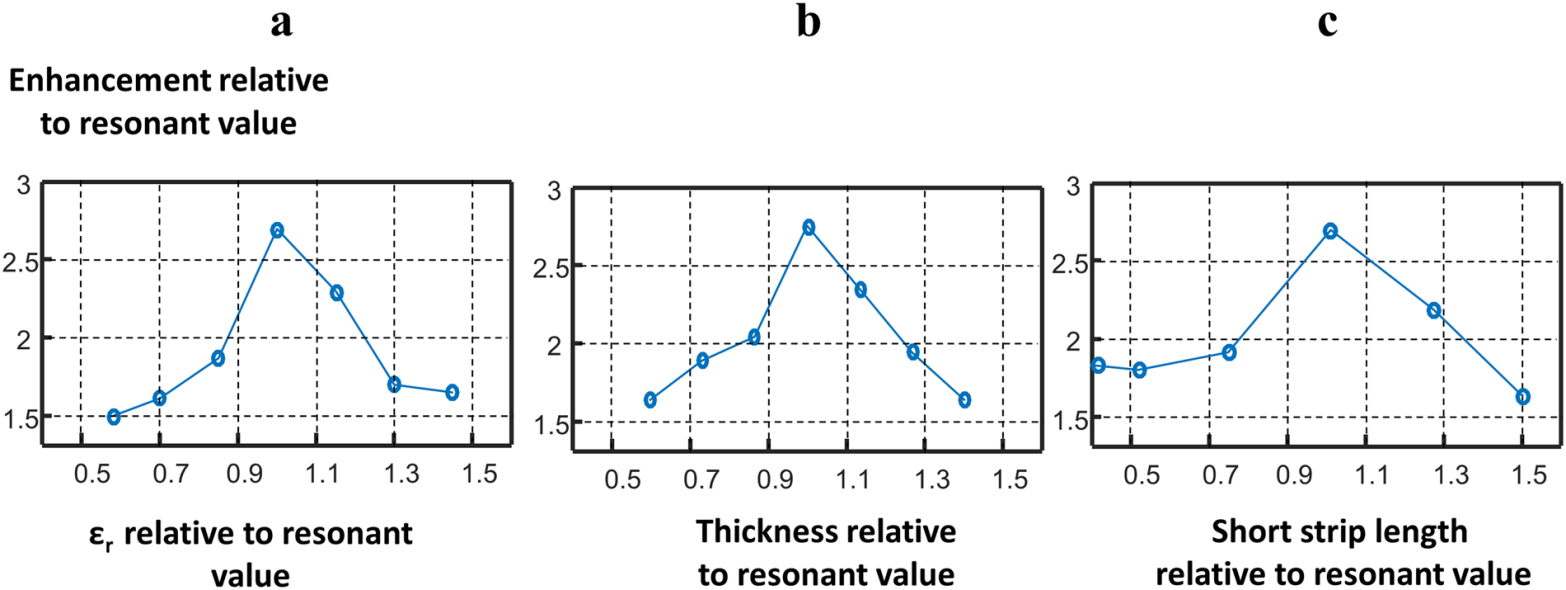
Parametric-dependence plots of the maximum enhancement ratio (averaged over a ROI of 6 × 6 mm) versus the value at resonance: (**a**) relative permittivity of the dielectric substrate, (**b**) thickness of the dielectric substrate and (**c**) length of the shorter strips.

Finally, the metasurface described in this paper has been specifically designed for the maximum increase in transmit and receive sensitivity close to the metasurface. However, the geometry of the metasurface has enormous flexibility in terms of tailoring the ability to “tune” its properties. For example, in many applications it might be more useful to have a lower enhancement factor which could be used in areas of the brain such as the temporal lobe which are associated with reduced transmit efficiency due to wavelength effects. Lower enhancement factors can be achieved by altering the geometry of the conducting elements in the metasurface. Figure 5 shows plots of the simulated enhancement as a function of the thickness and permittivity of the substrate, as well as the length of the short strips.

## Methods

The structure was constructed from 25 micrometre thick copper strips. The setup included long strips of 17.5 cm and a 3 × 3 matrix of short strips (3 cm length). The distance between the strips was 1 cm. The full structure size was 17.5 × 17.5 × 0.9 cm^3^ including a 0.8 cm thick dielectric layer and a plastic sheet on which copper strips were attached. The high permittivity dielectric layer consists of an aqueous suspension of calcium titanate in water, with a relative permittivity of 110 and conductivity of 0.09 S/m (CaTiO_3_ to water volume ratio of 3:1 v/v), which allowed a flexible structure to be formed. The metasurface was placed behind the head to increase the efficiency and sensitivity in the visual cortex.

3D EM simulations were performed using finite integration technique (FIT) software (CST Microwave Studio, Darmstadt, Germany). The RF transmit field is defined as the left circularly polarized transverse field *B*_1_^+^ = (*B*_1*x*_ + *B*_1*y*_)/*2* and the receive field as *B*_*1*_^*−*^ = (*B*_*1x*_ − *B*_*1y*_)/*2*. The magnitude of the RF transmit field defines the excitation tip angle θ applied to the spins in the excited volume, *θ* = *γB*_*1*_^+^ *τ* where γ is the gyromagnetic ratio and τ is the pulse duration. The signal-to-noise (SNR) of the image is proportional to sin(θ) ∙ B_1_^−*^/√P^37^, where P is the accepted power of the coil. All RF transmit (B_1_^+^) maps were normalized to an accepted power of 1 Watt. The simulation setup included a 16-rung high pass quadrature birdcage coil (inner diameter 30 cm; rung length 18 cm), corresponding to the transmit coil used for experimental measurements. For *in vivo* simulations the coil was loaded with the Virtual family model”Ella” dataset which consists of 76 different tissues with assigned values of permittivity and conductivity^38^. The mesh resolution was 1.0 × 1.0 × 1.0 mm^3^. The phantom setup simulations used a rectangular shape oil phantom with either a flat metasurface structure or a high permittivity pad placed on top. In the *in vivo* simulations, the metasurface structure was curved to best fit the shape of the head. To account for the actual structure of the metasurface, in addition to the high permittivity layer, the simulation also included an interface between the copper strips and the dielectric layer of 0.5 mm thickness with ε_r_ = 1.

Phantom and *in-vivo* images of a volunteer were acquired on a Philips Achieva 7 T MRI system. All methods were carried out in accordance with Leiden University Medical Center guidelines and regulations. All experimental protocols were approved by the the Leiden University Medical Centre Medical Ethics Committee. *In vivo* images and spectroscopy of the brain were acquired from healthy volunteers after informed consent was obtained, in accordance with the guidelines of the Leiden University Medical Centre Medical Ethics Committee. Phantom experiments were performed using a quadrature head birdcage coil (Nova Medical NM-008A-7P) for both transmit and receive. *In-vivo* brain imaging was acquired using a quadrature birdcage for RF transmit and close fitting 32-channel receive head coil (Nova Medical NMSC-025-32-7P). The images included a standard gradient-echo sequence that was used for SNR estimation and B_1_^+^ maps images were acquired using the DREAM^39^ sequence. The following scan parameters were used for gradient echo sequence: FOV 24 × 24 cm^2^, spatial resolution 1.5 × 1.5 × 5.0 mm^3^, TR/TE 10/3.4 ms, flip angle 5°; and for DREAM sequence: FOV 24 × 24 cm^2^, spatial resolution 2.5 × 2.5 × 5.0 mm^3^, TR/TE 3/1.7 ms, B_1_^+^ encoding tip angle 50° and imaging tip angle 10°. The localized ^1^H spectroscopy used STEAM sequence with TE of 12 ms, mixing time of 13 ms and TR of 3000 ms, 15 × 15 × 15 mm^3^ voxel, 64 averages.

## Electronic supplementary material

Supporting Information
