# Supplementary material for: Flexible and compact hybrid metasurfaces for enhanced ultra high field in vivo magnetic resonance imaging
Source: Sci Rep. 2017 May 10;7:1678. doi: 10.1038/s41598-017-01932-9 (PMC5431866; doi:10.1038/s41598-017-01932-9)
Supplement: Supplementary file 1 — Supporting Information [file 41598_2017_1932_MOESM1_ESM.pdf]

Supporting Information

Flexible and compact hybrid metasurfaces for enhanced ultra high field in vivo magnetic resonance imaging

*Rita Schmidt, Alexey Slobozhanyuk, Pavel Belov and Andrew Webb**

S1. Characteristics of first eigenmode of the metasurface structure used for MRI

The metasurface supports many different electromagnetic modes, which have different resonance frequencies and spatial distributions of magnetic and electric fields. The first (lowest frequency) eigenmode produces a magnetic field that is perpendicular to the structure. Figure S1 shows the spatial characteristics of this eigenmode for (a) a flat and (b) a curved metasurface used in this work.

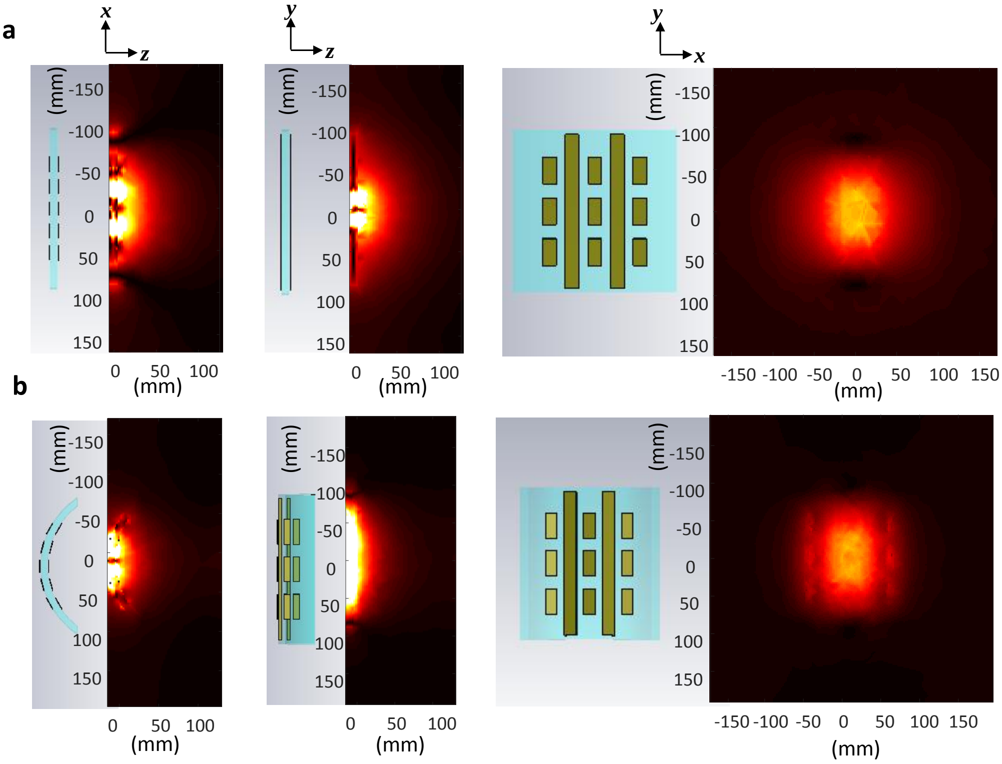

Figure S1 : Simulated first eigenmode of the metasurface structure. a |H| distribution maps for a flat metasurface. b, |H| maps for a curved metasurface. |H| maps are shown in three planes: the xz plane at y=0, the yz plane at z=0 and the xy plane at z=29. The cross-section of the structure is shown for each of these planes. The H_z component in all cases is equal to zero.

S2. The role of the matrix of short strips within the metasurface structure

The metasurface designed in this study includes sets of long and short strips. The dimensions and spacing of the long strips (in combination with the dielectric substrate) largely determine the resonant frequency of the desired eigenmode. If a matrix of shorter strips is incorporated into this assembly it enables additional flexibility in terms of shaping the local magnetic field enhancement. We performed EM simulations comparing the metasurface with and without the short strips: in each case the structure was tuned to the 7 T operating frequency. The simulation included transmit coil, metasurface and the brain model. Figure S2 illustrates that the magnetic field enhancement is greater with the short strips in place, as well as mitigating the area of low transmit efficiency along the central axis.

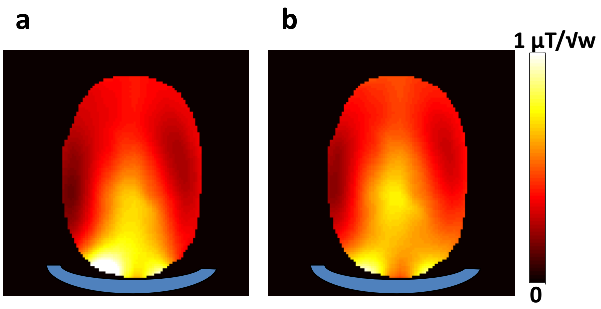

Figure S2: EM simulations using a metasurface with and without a matrix of short strips. The images show B_1^+ maps in the central axial plane. The metasurface used in (a) is the one described in the Methods section. The metasurface in (b) does not include the short strips (in order to tune the structure for the 7 T frequency, the long strips were shortened to 15.5 cm length). The maximum enhancement for case (a) is 2.7 versus 2.0 for case (b).

S3. Setup for the phantom experiment

Figure S3 shows the experimental setup used for the phantom experiments.

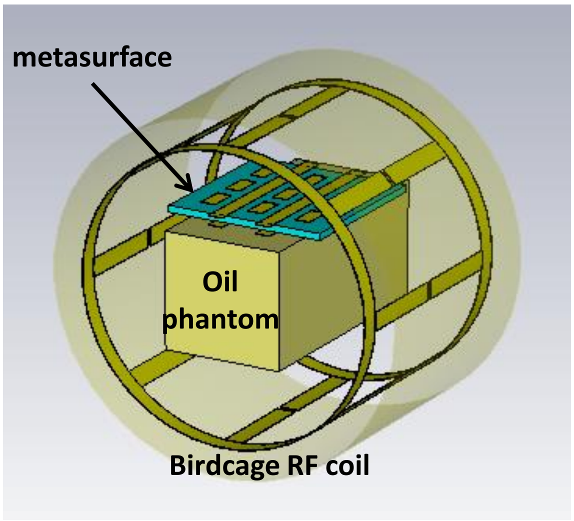

Figure S3: A schematic of the setup for the phantom experiment for which results are shown in the main text in Figure 2. The setup included an oil phantom, the metasurface placed flat on top of the phantom, and a quadrature birdcage RF coil that was used for both transmit and receive.

S4. Deviation in the resonant mode due to the bending of the metasurface structure

The frequency and the field distribution of the resonant mode generated by the metasurface depends on dimensions and spacing of the sub-units. Bending the metasurface can affect the resonant mode as has been demonstrated in previous work[1-3]. We analyzed changes in the characteristics of the resonant mode due to the bending of the structure by comparing a flat metasurface and one which was curved to fit the human model "Ella". Figure S4 shows the H-fields and E-fields with the metamaterial loaded with a phantom with relative permittivity 45, representing brain tissue at 7 T. The comparison show a slight difference in frequency of approximately 5 MHz and an increase of 3% in the peak of H-field and 7% in peak E-field in the curved metasurface. These results show that there is very little difference in the design procedure for metasurfaces with different desired curvature, and fine tuning can easily be performed.

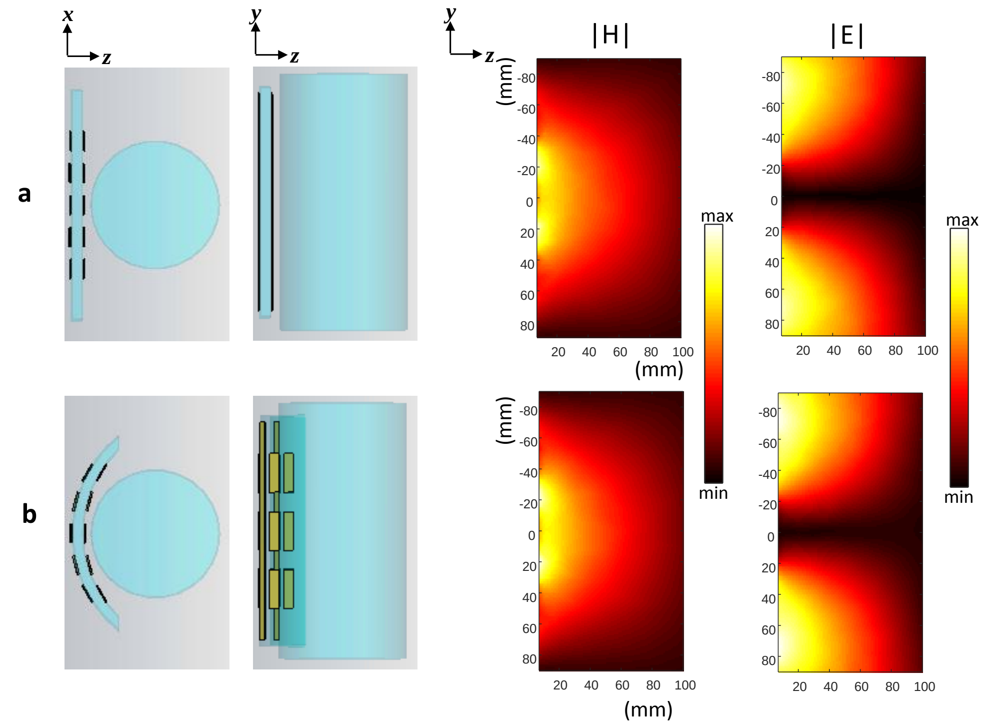

Figure S4 : Comparison of a flat and a curved metasurface structure. a, flat structure, b, curved structure. Cross-sections in xz and yz, and |H| and |E| maps in the yz plane are shown. The |H| maps of both cases have the same scaling, showing a 3% increase in the peak value for the curved setup. The |E| maps show a 7% increase in the peak value for the curved setup. The cylindrical phantom had a radius = 5 cm and length = 20 cm, and relative permittivity of 45. Simulations used an eigenmode solver and so the intensities of the H- and E-fields are in arbitrary units.

S5. Effect of the exact positioning of the metasurface on the enhancement factor.

In a realistic scanning environment, there is the chance of slight differences in the positioning of the metasurface due to the fact that different patients have different head sizes and ellipticity. In order to estimate the effects that these effects might have, we simulated one case with a 10 mm shift (to the right of the desired central location, and a second case where the eccentricity of the pseudo-elliptical head was increased by 10%. The results show that there is still a considerable signal enhancement, but this is reduced by mispositioning and a more elliptical head. It is certainly possible to make a larger metasurface which should make the enhancement less sensitive to such geometric differences.

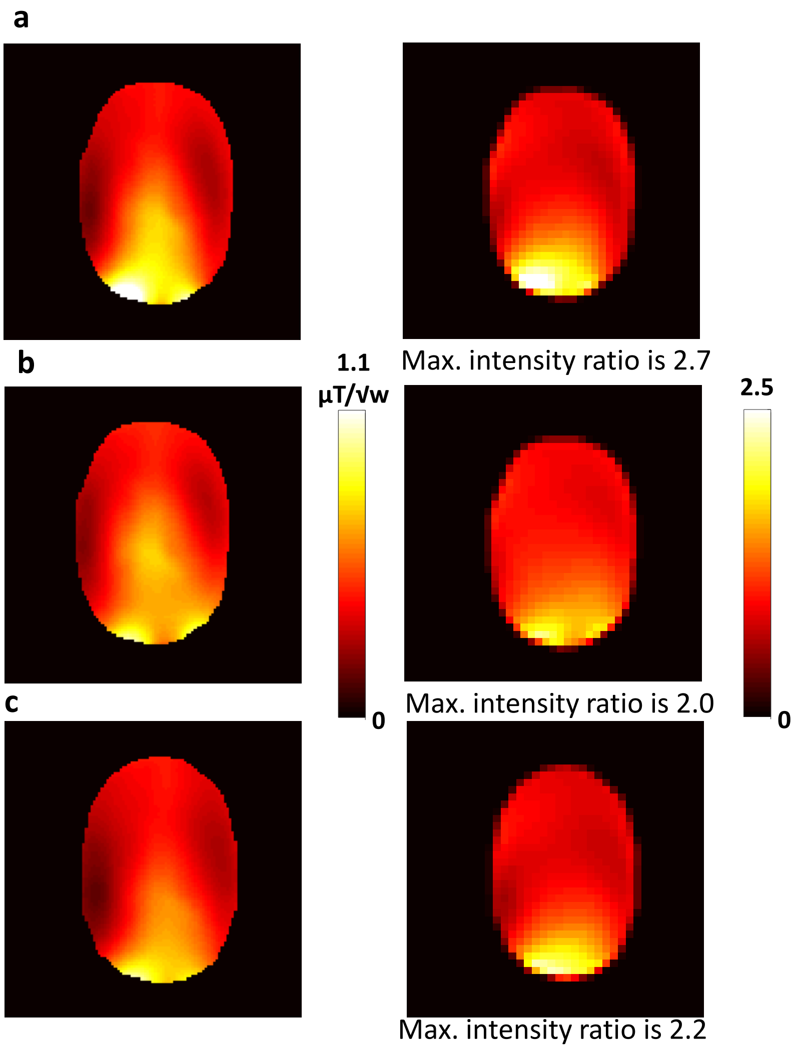

Figure S5: EM simulations showing the effect of different geometries on the enhancement factor. **(a)** The setup shown in Figure 3 with the metasurface centered around the region of interest. **(b)** The metasurface shifted to the right by 10 mm. **(c)** The ellipticity of the head increased by 10% The images show B_1^+ maps on the left and the enhancement ratio of the B_1^+ map compared to the one acquired without the metasurface on the right. The enhancement ratio maps were calculated for a reduced spatial resolution of 6x6 mm^2 in order to eliminate local maxima.

S6. Effect of the conducting elements of the metasurface on the SNR

Two concerns of using conducting metal strips as part of the metasurface are the possible shielding effects which can cause local shading in the image, as well as a potential increase in the noise level. In order to investigate whether these effects are present, we performed phantom and in vivo MR scans with only the metal strips from the metasurfaces present. In this case the structure is far from resonance, and so essentially no local enhancement is expected. Figure S6 shows images with the metal strips present and absent. No differences in signal-to-noise were measured in any regions of the image.

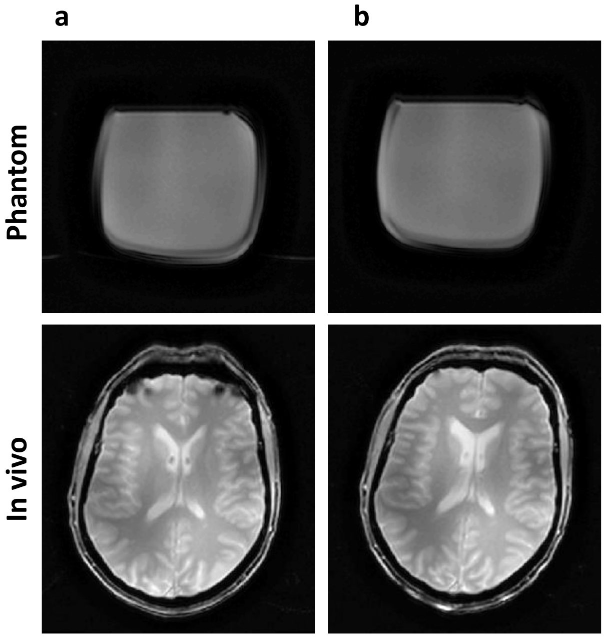

Figure S6: The effect of metallic strips on image quality. Images of an oil phantom (top) and in-vivo axial brain central slice (bottom) acquired without (a) and with (b) copper strips showed no difference in signal-to-noise.

References

1. Syms, R. R. A., Floume, T., Young, I. R., Solymar, L., & Rea, M.. Flexible magnetoinductive ring MRI detector: Design for invariant nearest-neighbour coupling. Metamaterials, 4(1), 1-14 (2010).
2. Lapine, M., Jelinek, L., Freire, M. J., & Marqués, R. Realistic metamaterial lenses: Limitations imposed by discrete structure. Physical Review B, 82(16), 165124 (2010).
3. Lihao, H., Huiling, Z., Zhang, H., & Quanming, C. Reduction of mutual coupling between closely-packed antenna elements with split ring resonator (SRR). In Microwave and Millimeter Wave Technology (ICMMT) 2010 International Conference IEEE, 1873-1875 (2010).
